# Activity-Induced Remodeling of Olfactory Bulb Microcircuits Revealed by Monosynaptic Tracing

**DOI:** 10.1371/journal.pone.0029423

**Published:** 2011-12-28

**Authors:** Benjamin R. Arenkiel, Hiroshi Hasegawa, Jason J. Yi, Rylan S. Larsen, Michael L. Wallace, Benjamin D. Philpot, Fan Wang, Michael D. Ehlers

**Affiliations:** 1 Department of Molecular and Human Genetics, Baylor College of Medicine, Houston, Texas, United States of America; 2 Jan and Dan Duncan Neurological Research Institute at Texas Children's Hospital, Houston, Texas, United States of America; 3 Department of Cell Biology, Duke University, Durham, North Carolina, United States of America; 4 Department of Neurobiology, Duke University, Durham, North Carolina, United States of America; 5 Department of Cell and Molecular Physiology, University of North Carolina, Chapel Hill, North Carolina, United States of America; 6 Curriculum in Neurobiology, University of North Carolina, Chapel Hill, North Carolina, United States of America; 7 UNC Neuroscience Center, University of North Carolina, Chapel Hill, North Carolina, United States of America; 8 Neurodevelopmental Disorders Research Center, University of North Carolina, Chapel Hill, North Carolina, United States of America; 9 Neuroscience Research Unit, Pfizer Global Research and Development, Groton, Connecticut, United States of America; 10 Department of Neuroscience, Baylor College of Medicine, Houston, Texas, United States of America; Columbia University, United States of America

## Abstract

The continued addition of new neurons to mature olfactory circuits represents a remarkable mode of cellular and structural brain plasticity. However, the anatomical configuration of newly established circuits, the types and numbers of neurons that form new synaptic connections, and the effect of sensory experience on synaptic connectivity in the olfactory bulb remain poorly understood. Using *in vivo* electroporation and monosynaptic tracing, we show that postnatal-born granule cells form synaptic connections with centrifugal inputs and mitral/tufted cells in the mouse olfactory bulb. In addition, newly born granule cells receive extensive input from local inhibitory short axon cells, a poorly understood cell population. The connectivity of short axon cells shows clustered organization, and their synaptic input onto newborn granule cells dramatically and selectively expands with odor stimulation. Our findings suggest that sensory experience promotes the synaptic integration of new neurons into cell type-specific olfactory circuits.

## Introduction

The mammalian brain ensures adaptive behavior through its large capacity for cellular and circuit plasticity. The diverse scales of neural plasticity range from single synapse modification [1]–[3] to network remodeling that accompanies ongoing neurogenesis [4]–[7]. Plasticity mechanisms accommodate changing environmental stimuli that are continuously relayed to the brain via multiple sensory modalities. Among sensory systems, the olfactory system possesses a large capacity for circuit plasticity through continued generation of new neurons in adult life. Such continuous incorporation of new neurons implies persistent, large-scale remodeling of synaptic connections, the nature of which is not well known.

Within the olfactory system, the axons of olfactory sensory neurons (OSNs) expressing the same odorant receptor [8] converge onto discrete glomeruli in the main olfactory bulb (MOB) [9], [10]. Organized around glomeruli, groups of mitral/tufted cells, as well as various interneurons, form connected networks that extend into all layers of the olfactory bulb [11]. These networks likely represent unitary modules for odor information processing [11]–[14] and may be functionally analogous to barrels in the somatosensory cortex or ocular dominance columns in the visual system.

The functional organization within and between MOB glomerular units has been the subject of intense investigation. Lateral interactions between glomeruli are mediated primarily by dendrodendritic synapses between mitral cells and granule cells [15]–[20], and the electrophysiological properties of these synapses have been well characterized [13], [21], [22]. Although mostly studied as singly recorded neurons or synaptically coupled pairs, these experiments support the notion that populations of neurons associated with multiple glomeruli are highly interconnected. Among the most studied forms of intrabulbar circuitry, granule cells provide inhibitory feedback onto spatially distant glomeruli by forming synapses with the lateral dendrites of mitral cells [13], [15]. In addition, synaptic inputs from both local short axon cells (SACs) and distant cortical neurons provide direct regulation of granule-mitral cell synapses [23]–[26]. Despite a central role in olfactory processing, the relative connectivity of individual granule cells to different cell types, the spatial organization of granule cell synaptic partners, and the regulation of granule cell connectivity by sensory stimulation remain unclear.

New GABAergic granule and periglomerular cells in the MOB are continually generated throughout adulthood [27]–[29]. Whereas many adult-born neurons fail to establish and maintain dendrodendritic synapses and ultimately undergo apoptosis [30]–[32], granule cells born during early stages of postnatal development tend to be long-lived and form stable synaptic connections [33]. We thus sought to define the patterns of cellular connectivity formed by postnatal-born granule cells in the MOB and determine how new granule cell microcircuits are influenced by sensory input. In the present study we employed monosynaptic circuit tracing using pseudotyped rabies virus together with a conditional red-fluorescence mouse reporter strain to label newborn olfactory bulb interneurons and their presynaptic partners *in vivo* [34]. We show that postnatal-born granule cells make synaptic connections with cortical inputs and multiple olfactory bulb cell types. The pattern of monosynaptic connectivity shows a clustered organization that is characterized by extensive presynaptic inputs from anatomically distinct short axon cells. Moreover, increased sensory experience by odor enrichment enhances SAC connectivity onto postnatal-born granule neurons. These results define the presynaptic repertoire of novel inputs onto newborn granule cells, and support a model whereby clustered patterns of organization in the olfactory bulb extend from local short axon cells to cohorts of deep granule cells that span the laminae of the olfactory bulb. The identification of numerous short axon cells presynaptic to new granule cells reveals unanticipated cellular interactions that occur during granule cell (GC) synapse development. Experience-driven changes in SAC-GC connectivity provides a circuit basis for refining or remodeling synaptic inputs upon exposure to a complex sensory environment through ongoing neurogenesis.

## Results

### Monosynaptic Tracing Reveals Synaptic Connectivity Onto Postnatal-Born Granule Cells

**Figure 1:**
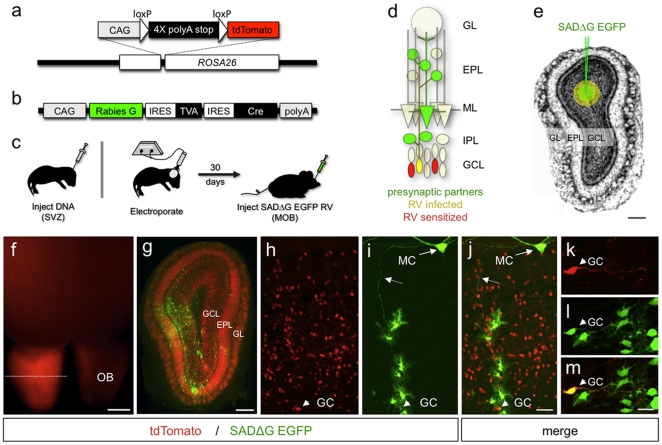
Engineered Rabies Virus Allows Monosynaptic Circuit Tracing in Olfactory Bulb. (a) Illustration of the *ROSA26-stop^flox^-tdTomato* conditional knock-in allele. (b) Illustration of the tri-cistronic G-IRES-TVA-IRES-Cre expression construct used for electroporation into the subventricular zone (SVZ) of *ROSA26-stop^flox^-tdTomato* mice. (c) Illustration of the *in vivo* electroporation procedure. Left, plasmid DNA encoding G-IRES-TVA-IRES-Cre was injected into the lateral ventricle of newborn mice. Middle, a square-pulse voltage was applied across the head to introduce the expression construct into SVZ progenitors. Right, 30 d following electroporation, pseudotyped SADΔG-EGFP RV was injected into the olfactory bulb for targeted infection of electroporated granule cells. (d) Diagram showing how pre- and postsynaptic neurons can be identified by two-color monosynaptic viral tracing in the conditional *ROSA26-stop^flox^-tdTomato* background. Red cells represent conditional tdTomato reporter expression following G-IRES-TVA-IRES-Cre electroporation. Yellow cells represent electroporated cells that are also infected by SADΔG-EGFP RV. Green cells represent presynaptic targets of the infected granule cells. GL, glomerular layer; EPL, external plexiform layer; ML, mitral cell layer; IPL, internal plexiform layer; GCL, granule cell layer. (e) Schematic illustrating the estimated domain of SADΔG-EGFP RV infection with respect to the entire bulb. On average, infected granule cells were detected within 302 µm of the injection site (green circle)±214 µm (yellow outer circle). Scale bar, 300 µm. (f) Whole mount view of *ROSA26-stop^flox^-tdTomato* mouse brain 30 d after unilateral G-IRES-TVA-IRES-Cre electroporation. Dashed line represents the coronal section imaged in (g)–(i). OB, olfactory bulb. Scale bar, 1 mm. (g) Coronal section through an electroporated and infected olfactory bulb showing tdTomato and SADΔG-EGFP expression. Scale bar, 300 µm. (h) Coronal section through the olfactory bulb of electroporated mice showing tdTomato-expressing granule cells at higher magnification. (i) Section shown in (h) imaged for SADΔG-EGFP following RV infection. (j) Merged view of (h) and (i). MC, mitral cell; GC, granule cell origin; dashed arrow, granule cell dendrite. Scale bar, 25 µm. (k)–(m) Dual tdTomato plus SADΔG-EGFP reporter expression, identifying a local granule cell microcircuit. (k) tdTomato expression in a single granule cell. (l) SADΔG-EGFP expression in the same ‘source’ granule cell shown in (k) and local presynaptic partners. (m) Merged reporter expression delineating source cell (yellow) and the local presynaptic partners (green). Short arrows in (k–m) point to the source granule cell. Scale bar, 10 µm.

Functional wiring in the olfactory bulb begins during embryogenesis and continues throughout postnatal life. Whereas embryonically derived interneurons of the MOB originate from the lateral ganglionic eminance and dorsal telencephalon [35]–[38], those born postnatally are generated exclusively from the subventricular zone (SVZ) of the lateral ventricle [39]–[42]. Although much is known about the cellular patterns of MOB interneuron development [35], [43], [44], the patterns of synaptic connectivity onto new granule cells are poorly understood. To determine the postnatal patterns of synaptic connectivity onto newborn granule cells, we performed *in vivo* monosynaptic circuit tracing [34]. For this we generated a conditional reporter mouse harboring a Cre/loxP-dependent allele (Figs. 1a, S1a, and S1b) capable of driving high levels of cytosolic tdTomato expression upon introduction of the plasmid G-IRES-TVA-IRES-Cre (Fig. 1b) that encodes components for targeted rabies virus (RV) infection, monosynaptic virus propagation, and Cre-mediated conditional reporter activation. In this design, neurons are genetically targeted for infection by expression of the TVA receptor, which binds selectively to RV particles pseudotyped with EnvA coat proteins [45]. Since the engineered RV mutant lacks the G coat protein, plasmid expression of the wildtype rabies-G coat protein enables precisely one round of “live” virus packaging and trans-synaptic infection of presynaptic cells [34], [46]–[50]. Retrograde spread of virus is strictly monosynaptic since only cells that receive G-IRES-TVA-IRES-Cre are capable of synthesizing live virus, whereas presynaptic targets do not contain the G protein and thus are incapable of producing infective RV particles. Replacement of the viral gene encoding the G capsid protein with an EGFP reporter renders presynaptic partners of RV-targeted cells brightly labeled [34], [51].

To test the functionality of our tracing system, we injected the G-IRES-TVA-IRES-Cre tri-cistronic construct into the lateral ventricles of embryonic day 14.5 (E14.5) *ROSA26-stop^flox^-tdTomato* mice, electroporated neuronal progenitors in the ventricular zone, and made *ex vivo* cortical slice cultures [52] (Figs. S1c–i). After two days, EnvA-pseudotyped SADΔG-EGFP RV particles [34] were applied to cultured brain slices to infect neurons expressing G-IRES-TVA-IRES-Cre. Three days later, we observed widespread tdTomato expression throughout layers 4 and 5 and in the walls of the ventricles (Figs. S1c, S1f, and S1i), indicating neurons expressing Cre recombinase via the G-IRES-TVA-IRES-Cre cassette. In addition, we observed EGFP expression in a small number of tdTomato-positive neurons directly infected by SADΔG-EGFP RV, which appeared yellow (Figs. S1h and S1i). Finally, we observed a large cohort of EGFP-expressing neurons lacking tdTomato expression, showing trans-synaptic spread of SADΔG-EGFP RV to presynaptic partners of RV-infected cells (Figs. S1g–i).

After confirming that our tricistronic rabies G-IRES-TVA-IRES-Cre construct (Fig. 1b) functioned in conjunction with the *ROSA26-stop^flox^-tdTomato* mouse line (Fig. 1a), we implemented this model system to examine the synaptic connectivity formed onto postnatal-born granule cells in the olfactory bulb. To target newborn granule cells for RV infection and subsequent monosynaptic tracing, we injected the G-IRES-TVA-IRES-Cre construct into the SVZ of postnatal day 2 (P2) mice and applied an external voltage across the brain to electroporate neuronal progenitors (Fig. 1c, left) [53]. After 30 d, at which point many of the electroporated cells have migrated to the MOB and formed functional synaptic connections [44], [54], EnvA-pseudotyped SADΔG-EGFP rabies virus was injected into the granule cell layer of the olfactory bulb to infect newly incorporated neurons expressing G-IRES-TVA-IRES-Cre (Fig. 1c, right). This experimental paradigm allowed us to probe the monosynaptic connectivity onto postnatal-born granule cells by directly visualizing vital fluorescence in neurons susceptible to pseudotyped RV infection (red), those that became infected (red and green, thus yellow), and presynaptic target neurons (green) (Fig. 1d). To target the electroporated granule cells for infection and monosynaptic tracing, we injected 250 µl the SADΔG-EGFP rabies virus 750 µm below the surface of the olfactory bulb, midway from the anterior and posterior boundaries. Although the methods of *in vivo* electroporation and viral infection are variable, by counting the number of infected source cells and determining the average bulbar volume spanned by these cells, we estimated viral spread and infection to encompass a spherical domain 300±200 µm in diameter (Fig. 1e, n = 10 labeled bulbs from 10 mice). To count labeled source cells, we made 100 µm slices through the entire bulb and identified all GCs that expressed both EGFP and tdTomato (n = 20–25 slices each, with the middle 3–5 slices harboring most of the labeled cells). Occasionally we infected periglomerular cells, which are also continually generated from electroporated SVZ [43]. In these instances we observed labeling of the cell types that contribute to the spherical glomerular structures (data not shown). To avoid infecting newborn periglomerular cells, we included a small volume (∼20 nl) of mineral oil in the tip of the injection pipette to prevent exposure of the virus while passing through the superficial bulb layers en route to the granule cell layer.

One week after RV infection, we processed the olfactory bulb and imaged fixed sections for reporter expression. Similar to what we observed in cortical slice explants (Fig. S1c–i), *in vivo* conditional reporter activation and trans-synaptic viral labeling was robust, and showed distinct patterns of presynaptic labeling (Figs. 1f–m). Although we began to observe viral-mediated EGFP in presynaptic target cells by 3 d following infection, high and stable levels of labeling were routinely achieved by 7 d. By 14 d post-infection, numerous presynaptic neurons showed abnormal morphologies, likely due to the extremely high levels of reporter expression driven by the RV genome. Thus, for all subsequent experiments, we chose 7 d post infection to investigate our presynaptic labeling. Since electroporation relies on generating a voltage across the ventricular space, injected DNA reproducibly showed unilateral patterns of reporter expression in the brain (Fig. 1f). This phenomenon was most obvious when imaging the conditional expression of tdTomato following transient introduction of Cre into the SVZ, which triggered tdTomato expression in neuronal progenitors that remained active in all daughter cells born from an electroporated lineage (Fig. 1g and 1h). Although this approach resulted in clonal labeling of many newborn granule cells with tdTomato, we were able to small numbers of postnatal-born granule cells for SADΔG-EGFP RV infection (Fig. 1i and 1j). This approach allowed us to resolve distinct clusters of presynaptic targets to electroporated granule cells (Fig. 1k–m). Based on the sparse infection by SADΔG-EGFP RV, the lack of infection points in close vicinity to one another, and the absence of clonal sectors (unlike those observed in cortical slices, Fig. S1), we reasoned that only a very small number of electroporated cells showed stable integration of the electroporated G-IRES-TVA-IRES-Cre plasmid. To further test this, we performed monosynaptic labeling of postnatal born granule cell networks while marking neuronal lineages born after electroporation with bromodeoxyuridine (BrdU) (Fig. S2a). At 14 d after electroporation, mice were supplemented with BrdU in their drinking water for 2 weeks to label all neurons born after that time, followed by RV infection and subsequent tissue processing. By comparing the number of green presynaptic inputs to the number of BrdU labeled green cells, we rarely ever observed newborn neurons that were doubly labeled (Fig. S2b–e). These data show that only a very small fraction of the tdTomato marked cells harbor the monosynaptic tracing components, and suggest that stable integration of our expression plasmid is rare and likely occurs in postmitotic neuronal precursors. By separately labeling primary points of infection (yellow source cells) and the cohort of neurons that provide their presynaptic input (green) (Fig. 1d), this modular virus-based molecular genetic system thus allowed us to directly visualize the synaptic microcircuits associated with postnatal-born granule cells.

### Cohorts of Mitral Cells and Short Axon Cells Synapse Onto New Granule Cells

**Figure 2:**
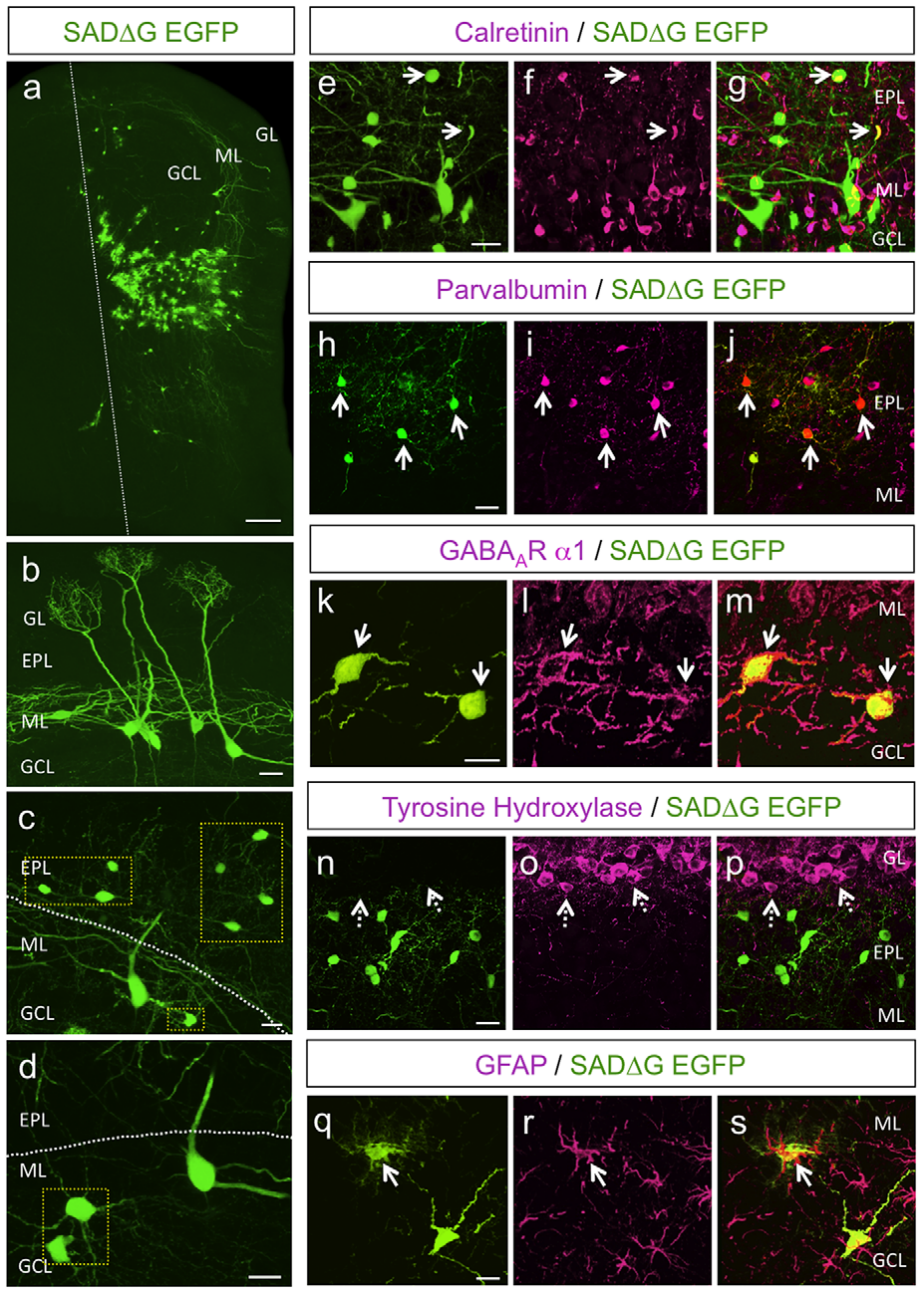
SADΔG-EGFP RV is Retrogradely Transported from Granule Cells to Presynaptic Targets in Olfactory Bulb. (a) A coronal section of olfactory bulb following monosynaptic viral tracing showing a clustered pattern of presynaptic labeling. The dashed line shows the midline through a coronal section of MOB. Scale bar, 100 µm. (b) SADΔG-EGFP expression in presynaptic mitral cells. Scale bar, 25 µm. (c)–(d) SADΔG-EGFP expression in short axon cells in the external plexiform and granule cell layers (hatched yellow boxes). Scale bars, 20 µm. (e)–(g) Partial colabeling of calretinin in SADΔG-EGFP expressing cells. Scale bar, 20 µm. (h)–(j) Partial colabeling of parvalbumin in SADΔG-EGFP expressing cells. Scale bar, 20 µm. (k)–(m) Overlapping expression of GABA_A_ R α1 in SADΔG-EGFP labeled short axon cells. Scale bar, 15 µm. (n)–(p) SADΔG-EGFP expressing cells do not express tyrosine hydroxylase, and thus are not periglomerular. Dashed arrows point to a tyrosine hydroxylase positive cell not labeled by SADΔG-EGFP RV. Scale bar, 20 µm. (q)–(s) Expression of GFAP in occasional SADΔG-EGFP labeled glial cells. Scale bar, 10 µm. Arrows point to overlapping marker expression in SADΔG-EGFP labeled cells. For all panels, GL, glomerular layer; EPL, external plexiform layer; ML, mitral cell layer; GCL, granule cell layer.

The full complement of neurons that contact newly integrated granule cells is unknown. In addition to dendrodendritic synapses formed between mitral/tufted cells and granule cells, it has recently been shown that granule cells form functional connections with multiple local interneuron cell types [24], [26]. After genetically targeting postnatal granule cells for SADΔG-EGFP RV infection, we observed extensive labeling throughout the granule cell, mitral cell, and external plexiform layers (EPL) in neurons presynaptic to electroporated granule cells. Interestingly, we noted clustered patterns of connectivity (Fig. 2a). These clustered networks contained mitral cells [55] (Fig. 2b) and other inframitral layer cells that morphologically corresponded to both superficial and deep short axon cells (SACs) (Figs. 2c and 2d). By restricting rabies-G, TVA, and Cre expression to postnatal-born interneurons through timed *in vivo* electroporation, we never observed conditional tdTomato reporter expression in cell types other than periglomerular and granule cells. Further, we never observed trans-synaptic labeling between granule cells born after electroporation (Fig. S2 and data not shown), arguing against nonspecific RV uptake through proximity spillover.

Varying the amount of pseudotyped RV that was injected into the MOB allowed us to control the number of virally infected source cells. Levels of viral infection ranged from very low (100 nl) (Figs. 1k–m) to high (500 nl) (Fig. 2a), and thus could be adjusted for optimal microcircuit analysis. For our experiments, we used conditions (250 nl) that achieved a moderate level of labeling (see Fig. 1e and Experimental Procedures) to facilitate source cell and presynaptic partner identification. To validate the retrograde trans-synaptic transport of SADΔG-EGFP RV, we examined EGFP expression in cortical brain regions known to send long-range centrifugal inputs to the MOB and make synapses onto granule cells [13], [56]. Consistent with the known presynaptic transport of RV, we observed strong SADΔG-EGFP expression in neurons of the anterior olfactory nucleus (AON), horizontal limb of the diagonal band nucleus (HDB), and piriform cortex (Fig. S3). Moreover, a complete absence of EGFP label in olfactory sensory neurons (OSNs), which heavily innervate the MOB and synapse onto mitral cells, confirmed that viral labeling stops after one presynaptic connection (data not shown). Together these data show that postnatal-born granule cells can be precisely targeted for RV infection, demonstrate both short- and long-range synaptic connectivity onto postnatal-born granule cells, and reveal clustered patterns of organization intrinsic to granule cell microcircuits.

To further investigate the repertoire of cells presynaptic to postnatal born granule cells, we performed immunohistochemical analysis on thin sections of MOB and counted the number of EGFP-positive, RV-infected neurons that were negative for tdTomato (hence, presynaptic to Cre-expressing granule cells) using molecular markers expressed by olfactory bulb cell types [57]. To begin to elucidate the molecular identity of the cells presynaptic to newborn granule cells, we made 50 µm sections through the labeled domain of the MOB (average 6 sections per bulb), performed immunohistochemistry, and counted EGFP labeled cells to determine the percentage of cells that showed overlapping expression of various interneuron markers. Whereas only a subset of EGFP-positive cells expressed the interneuron markers calretinin (46±5% EGFP-positive cells, n = 100 cells in 18 sections from 4 mice; Figs. 2e–g) and parvalbumin (55±7%; Figs. 2h–j), we routinely observed co-expression of the GABA_A_ receptor subunit α1 (84±4%; Figs. 2k–m), which is highly and selectively expressed in SACs residing within inframitral cell layers [57]. Thus, through immunohistochemical analysis against interneuron markers in the olfactory bulb, ∼50% of the SADΔG-EGFP labeled cells expressed parvalbumin and/or calretinin, whereas >80% of the local presynaptic inputs expressed GABA_A_ receptor subunit α1. We never detected SADΔG-EGFP expression in cell types that express tyrosine hydroxylase in the upper EPL or glomerular cell layer (Figs. 2n–p). Interestingly, we occasionally observed SADΔG-EGFP expression in proximate glial cells that were directly contacting source granule cell (GC) dendrites, as shown by colocalization with glial fibrillary acidic protein (GFAP) (Figs. 2q–s), suggesting a potential role for glial contact in synapse remodeling of newborn granule cells [58]–[60]. Together, these data show that, in addition to forming well-described contacts with centrifugal inputs and dendrodendritic synapses with mitral and tufted cells, postnatal-born granule cells receive extensive input from local SACs in the MOB.

To more closely examine the connectivity made between granule cells and their presynaptic partners, we took advantage of the dual-color labeling scheme of our experimental system to count the number and types of neurons that were labeled with SADΔG-EGFP RV by monosynaptic transfer. Given that all granule cells targeted for G-IRES-TVA-IRES-Cre electroporation express tdTomato (red), and only a subset of those cells became infected by injected SADΔG-EGFP RV (red and green, thus yellow), presynaptic targets can be clearly identified by the presence of only EGFP (green) (Fig. 1d). From our immunohistochemical data identifying the majority of non-mitral cell presynaptic neurons as SACs (Figs. 2c and 2d), we sought to determine the relative connectivity of SACs onto new granule cells. We counted doubly labeled (yellow) granule source cells and all of their green presynaptic neuronal partners in 100 µm thick serial sections of entire olfactory bulbs. On average we identified 33±8.1 doubly labeled source cells per bulb. Interestingly, we found that the ratio of presynaptically labeled short axon cells to RV-infected granule cells was quite high (4.6±0.8, SEM, n = 7 olfactory bulbs), revealing a previously unappreciated level of local SAC input to newborn granule cells.

**Figure 3:**
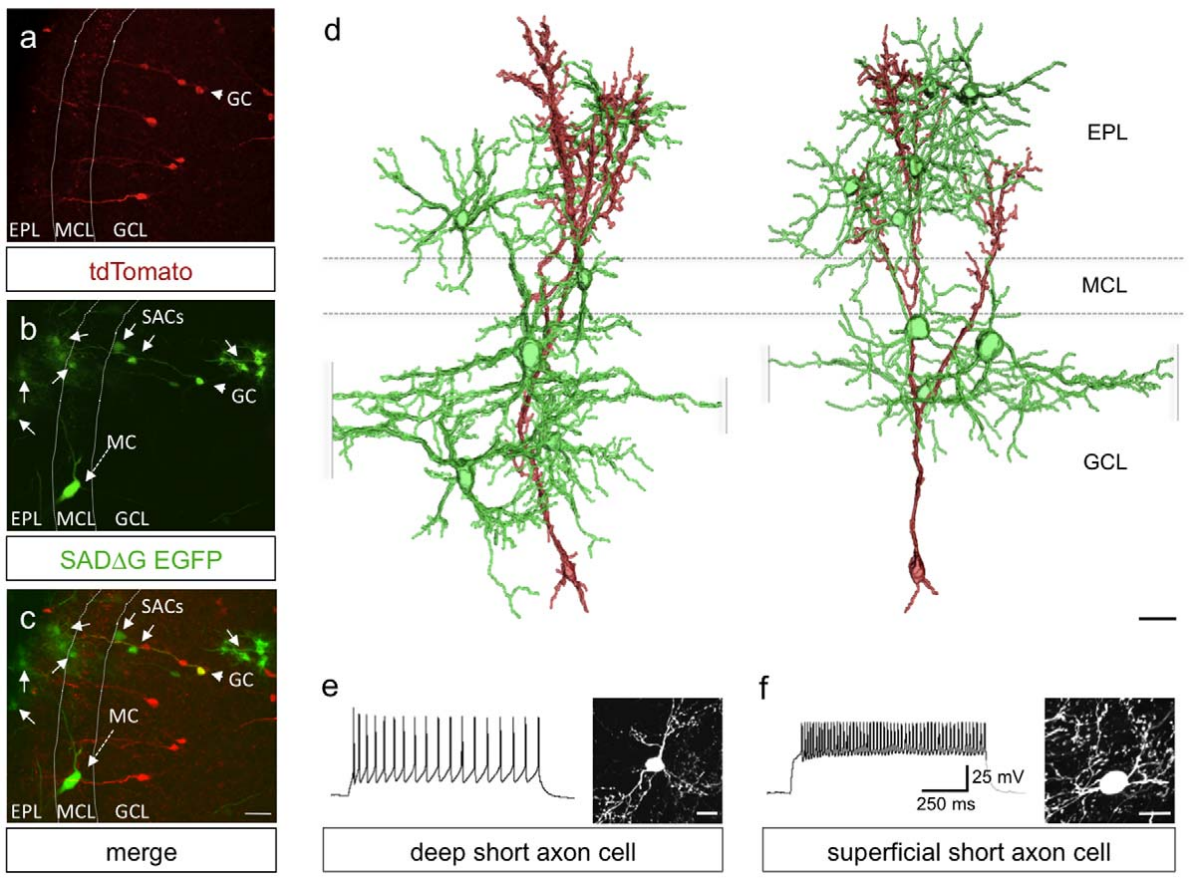
Newborn Granule Cells Receive Extensive Input from Short Axon Cells. (a)–(c) An SADΔG-EGFP labeled olfactory bulb microcircuit in which pre- and postsynaptic cell types can be identified by differential reporter expression. Electroporated cells appear red due to Cre activation of tdTomato expression. SADΔG-EGFP infected source cells appear yellow due to co-expression of tdTomato and EGFP. Presynaptic partners become trans-synaptically infected with SADΔG-EGFP but lack Cre and thus appear green. The dashed arrow points to a mitral cell, MC. Arrows point to short axon cells, SACs. Arrowheads indicate a source granule cell, GC. For (a)–(d): EPL, external plexiform layer; ML, mitral cell layer; GCL, granule cell layer. (a) tdTomato expression (red) in recombined Cre-expressing granule cells. (b) SADΔG-EGFP expression in a granule source cell (arrowhead) and its presynaptic targets (arrows). (c) Merge of (a) and (b). Scale bar, 20 µm. (d) Examples of volume-rendered reconstructions showing local short axon cell microcircuits with synaptic contacts onto newborn granule cells. The postsynaptic granule cells are shown in red, and the presynaptic short axon cells are shown in green. Scale bar, 15 µm. (e)–(f) Examples of action potential responses to depolarizing current injection and images of the short axon cell types observed to make synaptic contacts onto newborn granule cells. Shown are firing responses (left) and cellular morphologies (right) of a representative deep short axon cell (e) and superficial short axon cell (f) with contacts onto a newborn granule cell. Scale bars, 15 and 10 µm, respectively.

We next investigated the clustered architecture of SACs presynaptic to newly integrated GCs. For this, we selected olfactory bulb tissue that had relatively sparse SADΔG-EGFP RV infection and presynaptic labeling in order to facilitate the imaging of labeled SAC to GC microcircuits with cellular resolution (Figs. 3a–c). To determine the relative numbers, locations, and types of presynaptic neurons that contribute to the clustered network, we prepared semi-thick brain slices (150–200 µm) that were optically cleared in glycerol for z-stack confocal imaging and 3-dimensional volume rendering [61]. To quantify the inputs to granule source cells, we took serial image planes through the slices at 2 µm intervals, counting all doubly labeled and green-only neurons to determine the ratio of presynaptic inputs to granule source cells. In addition to clearly identifiable mitral cells (Fig. 2a–d), reconstructed image stacks revealed two main populations of SACs which have been previously characterized as deep SACs and superficial SACs [24], [57], [62]. Quantitative analysis revealed 5.6±1.5 SACs per resolvable clustered network (Fig. 3d), and an average width of the network (including SAC cell bodies and proximal dendrites) of 148.5±40.6 µm (n = 10 SAC/GC networks from 4 bulbs, of 4 mice ± SEM). Neurons presynaptic to individual GCs were categorized as SACs by morphology, molecular marker analysis, and electrophysiological properties (Fig. 2 and Fig. 3d–f), whereas the widths of GC-SAC networks were determined by the measuring the lengths of clearly resolvable SAC dendrites. Consistent with previous reports [24], [63], presynaptic deep SACs showed low frequency trains of action potentials with depolarizing current injections, whereas superficial SACs responded with high frequency patterns of firing (Fig. 3e–f). It is unlikely that our viral labeling identifies the full complement of functional synapses onto postnatal-born granule cells due to the unknown efficacy of particle transfer. In addition, it is likely that we underestimated the width of a functional MOB cluster by not including distal SAC axonal arbors in our characterization due to the lack of fine resolution imaging in thick brain slices. Altogether, these data reveal an unexpected and extensive local connectivity between SACs and newly integrated granule cells in olfactory bulb.

### Odor Enrichment Increases SAC-GC Connectivity in the Olfactory Bulb

**Figure 4:**
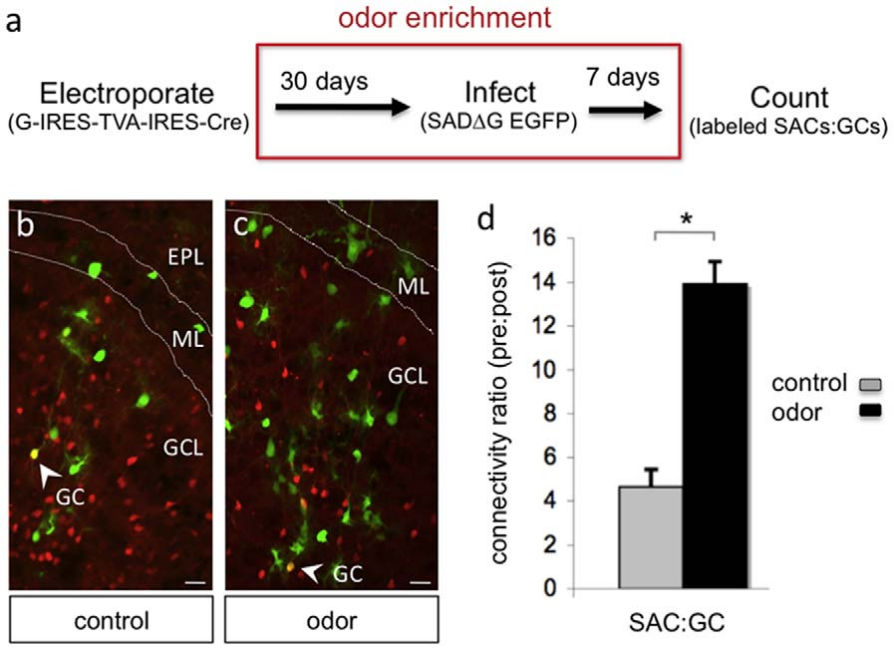
Odor Enrichment Increases SAC Connectivity onto Granule Cells. (a) Schematic of experimental paradigm to track changes in granule cell connectivity following odor enrichment. (b) Dual labeled region of a control olfactory bulb showing SADΔG-EGFP expression in presynaptic targets. Scale bar, 10 µm. (c) Dual labeled olfactory bulb from a mouse subjected to odor enrichment showing increased presynaptic labeling (green). Arrows points to source granule cells (GC) which appear yellow due to co-expression of tdTomato and EGFP. Presynaptic partners appear green. Scale bar, 10 µm. EPL, external plexiform layer; ML, mitral cell layer; GCL, granule cell layer. (d) Connectivity ratio between postsynaptic granule cells and presynaptic short axon cells (SAC∶GC) under control conditions or following odor enrichment. ^*^p<0.01, Student's t-test.

To examine how sensory experience influences granule cell microcircuits, we analyzed the patterns of connectivity between granule cells and their presynaptic targets following olfactory stimulation. We concentrated on synaptic input from SACs as these cells were robustly labeled using RV monosynaptic tracing (Figs. 2, 3). To provide a broad palette of odors for long-term sensory enrichment, we designed a robotic system for continuous cycled delivery of multiple odorants to freely exploring mice (see Fig. S4 and methods). For odor enrichment, *ROSA26-stop^flox^-tdTomato* mice were subjected to *in vivo* SVZ electroporation with G-IRES-TVA-IRES-Cre and reared with their mothers for 30 d in cages ported for odor delivery (Fig. S4). After the 30 d odor stimulation period, SADΔG-EGFP RV was injected into the granule cell layer of the olfactory bulb, and 7 d later the olfactory bulb was dissected and sectioned to count dual-labeled (yellow) granule cells and their single-labeled (green) presynaptic partners (Fig. 4a). Odor exposure induced a dramatic increase in the number of SACs presynaptically coupled to new granule cells compared to the non-odor enriched control group (Figs. 4b and 4c). Specifically, odor stimulation tripled the connectivity ratio of SACs onto source GCs (control, 4.6±0.8; odor-enriched, 13.8±1.0; n = 3 bulbs from 3 mice, ± SEM) (Fig. 4d). On average, we counted 48±14 doubly labeled source cells per bulb in mice subjected to odor enrichment. The increase in labeled presynaptic partners was not simply a reflection of an increased absolute number of new granule cells since the quantification normalizes to the number of labeled granule cells. Thus, odor enrichment enhances SAC connectivity onto new granule cells.

**Figure 5:**
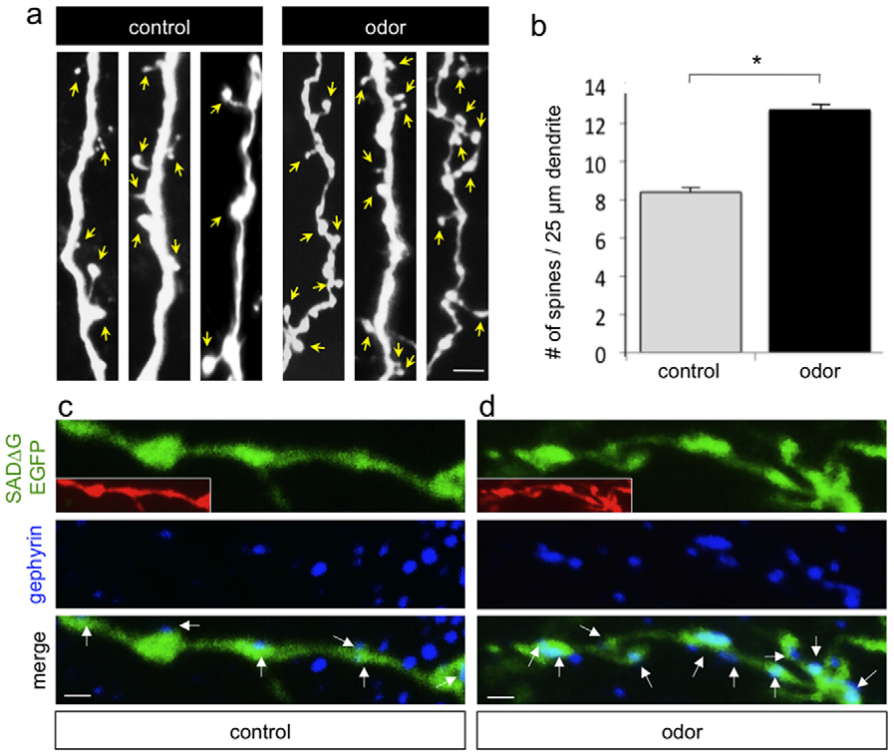
Odor Stimulation Increases Synaptic Inputs onto Newborn Granule Cells. (a) Dendrites from granule cells in control or odor exposed mice showing the increased number of spines following odor enrichment. Yellow arrows point to individual spines. Scale bar, 3 µm. (b) Data represent means ± SEM of spine number per 25 µm dendrite on granule cells in mice exposed to cycled odorants (odor) compared to non-odor exposed controls. ^*^p<0.001, Student's t-test. (c) Gephyrin labeling to reveal inhibitory GABAergic synapses on control granule cell dendrites. Insets in (c) and (d) show tdTomato expression in doubly labeled dendrites. Scale bars, 2 µm. (d) Increased number of gephyrin labeled inhibitory synapses contacting dendrites of granule cells from mice subjected to odor enrichment.

One possible explanation for the increased labeling of presynaptic cells upon odor stimulation is that elevated neuronal activity enhances RV transfer at existing synapses. To address this issue, we prepared olfactory bulb explants from mice that had been electroporated with a plasmid encoding the monosynaptic tracing components, infected with the SADΔG-EGFP RV, and cultured in the presence of pharmacological agents to block synaptic transmission. We found that blocking SNARE-dependent neurotransmitter release, action potentials, or fast glutamatergic neurotransmission had no significant effect on the number of monosynaptically labeled cells compared to untreated controls (Fig. S5). Thus, trans-synaptic transfer of RV is insensitive to activity manipulations over several days *in vitro*, and we conclude that the odor-induced expansion in granule cell circuit labeling *in vivo* (Figs. 4c and 4d) is attributable to changes in the number of synaptic inputs per granule cell, rather than to alterations in the effectiveness of RV transfer between neurons following olfactory stimulation. Consistent with this notion, we observed morphological differences in the granule cells themselves. Doubly labeled granule source cells in the bulbs of mice that were reared in odor enriched environments showed a significant increase in the number of dendritic protrusions (control, 8.4±0.7 per 25 µm dendrite, n = 17 doubly labeled neurons from 3 mice; odor, 12.7±0.9; n = 18 doubly labeled neurons from 4 mice; p<0.01; Figs. 5a and 5b). We also noted a significant increase in the number of inhibitory synapses on the dendrites of source granule cells by staining for the inhibitory synapse marker gephyrin (control, 7.8±0.9 gephyrin clusters per 35 µm dendrite; odor, 11.9±1.3; n = 12 neurons from 3 bulbs each; p<0.02; Figs. 5c and 5d). To restrict our analysis to synaptic changes in postnatal born neurons, gephyrin-positive puncta were only counted if they could be clearly colocalized within doubly labeled granule source cells. Corresponding to enhanced SAC input onto granule cells following odor enrichment, we also noted increased mitral cell input (data not shown). Given these findings, we cannot rule out that a portion of the gephyrin-positive puncta may indeed correspond to increased centrifugal input from other inhibitory cell types. Nonetheless, these data support increased presynaptic input onto newborn neurons following odor enrichment, and reveal activity-induced expansion of SAC circuitry.

**Figure 6:**
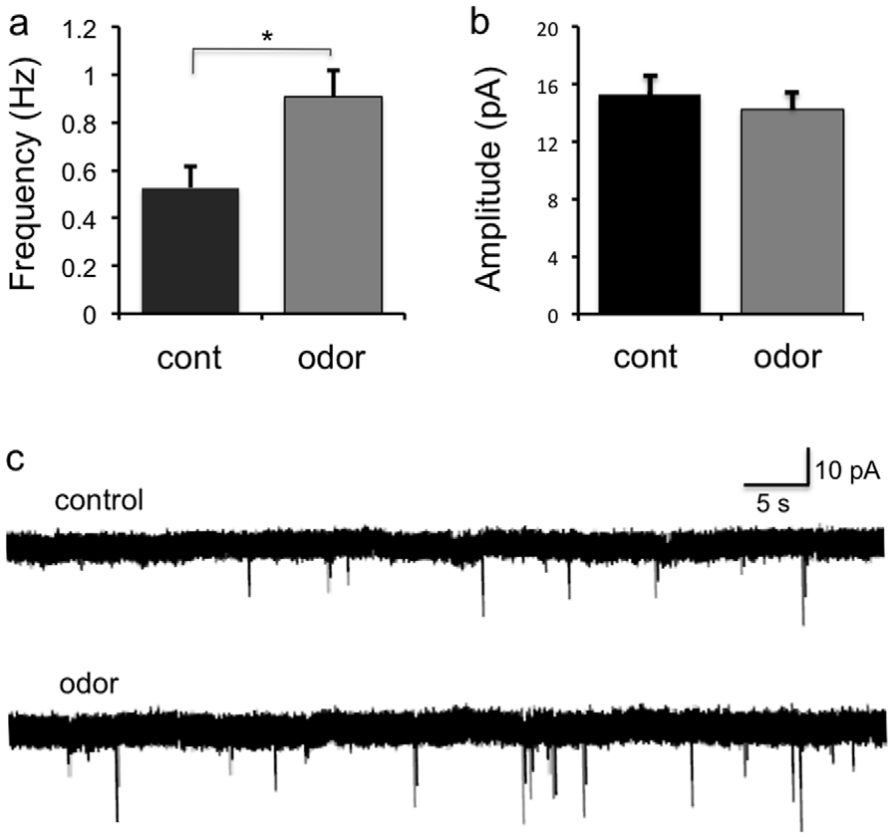
Odor Enrichment Increases Inhibitory Drive Onto Newborn Granule Cells. (a)–(b) Quantitative analysis of (a) average frequency and (b) amplitude of mIPSCs recorded from labeled granule cells in control and odor-enriched mice. Odor enrichment increased the frequency, but not amplitude, of granule cell mIPSCs (control, n = 9; odor, n = 10, ^*^p<0.01, unpaired t-test). (c) Representative voltage-clamp recordings of mIPSCs from granule cells in acute MOB slices from control and odor-enriched mice.

SACs provide GABAergic inhibitory input onto MOB granule cells [24], [26], [64]. We next tested whether the odor-induced increase in both RV-labeled SACs presynaptic to GCs and gephyrin-labeled inhibitory synapses onto GCs corresponds with changes in functional synaptic connectivity. To this end, we performed whole-cell patch clamp recordings in acute brain slices from labeled MOB granule cells following *in vivo* postnatal electoporation and measured miniature inhibitory postsynaptic currents (mIPSCs). Consistent with the observed increase in gephyrin puncta (Figs. 5c,d), odor stimulation significantly increased mIPSC frequency in postnatal born granule cells (Fig. 6). Whereas mIPSC amplitudes were similar between experimental groups (control, 15.2±1.4 pA; odor, 14.4±1.3 pA; Fig. 6b), mIPSC frequency was increased in odor-exposed animals (control, 0.55±0.06 Hz, n = 9 cells from 3 mice; odor, 0.89 Hz±0.07, n = 10 cells from 4 mice, p<0.01, unpaired t-test; Fig. 6a). Taken together, these data show that sensory experience expands connectivity between short axon cells and new granule cells, defining a novel cell type-specific reorganization of olfactory circuits in response to odor-induced activity.

## Discussion

In the present study we combined genetic circuit tracing and *in vivo* labeling technologies to map monosynaptic connections made between postnatal-born granule cells and their presynaptic input neurons in mouse olfactory bulb. We found that, in addition to previous known connections with mitral/tufted cells, postnatal-born granule cells show extensive connectivity to short axon cells, and these short axon cell inputs contribute to clustered architecture in the olfactory bulb. In addition, we have found that increased sensory experience in the form of odor stimulation expands circuit connectivity made onto newborn granule cells. This increase in synaptic connectivity manifests as a threefold expansion in presynaptically coupled short axon cells, and is accompanied by a corresponding increase of granule cell inhibitory synapses and mIPSCs, as well as changes in dendritic morphology.

### Short Axon Cell Input onto Granule Cells

Implementing a highly selective *in vivo* genetic targeting strategy, we investigated the synaptic patterns of connectivity that are made between postnatal-born granule cells and their presynaptic targets using engineered rabies virus for monosynaptic circuit tracing. Our experimental design enabled us to selectively target granule cells for rabies infection and propagation via *in vivo* electroporation [53], allowing direct determination of the cell types presynaptic to targeted granule cells. Using this approach, we observed discrete synaptic networks associated with granule cell microcircuits. Surprisingly, the majority of neurons making local presynaptic inputs onto postnatal-born granule cells were identified as SACs in the granule cell and external plexiform layers of the olfactory bulb.

Recent morphological and electrophysiological studies have shown that SACs elaborate axonal arborizations throughout all layers of the olfactory bulb, are presynaptic to resident granule cells, and provide GABAergic input that modulates granule cell firing [24], [26], [57]. Interestingly, the superficial SACs we have identified in this study display morphological and electrophysiological characteristics similar to Van Gehuchten neurons, which express many of the same molecular markers as deep SACs, but are thought to make contacts on both GCs and mitral cells, and are axon-less [62]–[66]. These properties suggest that SACs provide spatial refinement of local inhibitory circuits that sculpt the firing properties of mitral cells. In other brain areas, GABAergic input during neuronal development contributes to cell differentiation, survival, and circuit maturation [67]–[71]. Given ongoing adult neurogenesis, resident short axon cells in the mature olfactory bulb may similarly contribute to dynamic remodeling, differentiation, or survival of newly incorporated granule cells [43], [72]. Thus, in addition to providing inhibitory control over dendrodendritic synapses in the mature circuit, SACs may regulate synaptic integration of newborn granule cells through developmental signaling mechanisms. In such a scenario, odor enrichment would not only modify network activity in the bulb, but also promote GABAergic input onto new GCs from select cohorts of SACs. These odor networks may serve as hubs of activity that are more favorable or attractive for newborn neuron synapse formation and survival. More refined modes of circuit activity manipulations using cell type-specific chemical genetic or optogenetic manipulations will be required to fully address this notion [46].

Our experimental approach was effective in demarcating presynaptic cell types known to make functional connections onto postnatal-born granule cells, including centrifugal input from olfactory cortex, mitral/tufted cells, and SACs. The extensive connectivity made by SACs onto granule cells raises the question of the identity of neurons that are presynaptic to SACs. What cell types drive the inhibitory influence that SACs in turn relay onto granule cells? Although it has been proposed that mitral cells could serve this role [24], direct evidence in support of this model is lacking. Future genetically targeted monosynaptic tracing would be well suited to address this question. Another intriguing observation was the viral labeling of certain sparse glial cells found to be in direct physical contact with granule “source” cells. Such labeling could be due to uptake of viral particles at neuron-glia contacts. Indeed newborn neocortical neurons are known to form transient gap junction contacts onto radial glia [73], and functional neuron-glia synapses have been described [74], [75]. Alternatively, glial labeling could arise by selective engulfment or uptake of cell remnants during apoptosis or synapse pruning [76], [77]. It will be important for future studies to examine the electrophysiological and ultrastructural nature of this neuron-glia interaction.

### Odor Experience Expands Olfactory Bulb Circuitry

SACs are born during embryonic development and are resident prior to either sensory input or granule cell integration [35], [78]. Thus, the clustered organization onto granule cells may reflect developmental patterning events that occur during forebrain maturation, or alternatively may represent local connectivity that is elaborated or pruned in response to patterns of sensory experience or glomerular activity. In our experimental system, we observed clustered SAC inputs onto granule cells in both unstimulated controls and odor-enriched groups. However, in addition to the known enrichment of postnatal-born neurons that become integrated into olfactory bulb circuits following odor stimulation [7], [31], we have found that the number of neurons forming functional presynaptic inputs onto individual granule cells is also dramatically increased following odor enrichment. This increased level of presynaptic input onto postsynaptic granule source cells was revealed by greater numbers of labeled presynaptic targets, morphological changes in postsynaptic spine structures and inhibitory scaffolds, as well as increased inhibitory drive. The presence of more presynaptic inputs onto newborn GCs does not necessarily imply a linear increase in cell-to-cell connectivity. That is, in response to odor stimulation additional presynaptic cell types could form synaptic connections without an overall change in the absolute number of synapses onto a GC, provided that the overall connectivity ratio for any given presynaptic target cell decreases. Although the focus of our analysis was on the increased number of inputs onto GCs by SACs, we also observed an increase in the number of labeled presynaptic mitral cells (data not shown). It is likely that the changes we observe in cellular morphology and electrophysiological output in response to odor enrichment reflects a composite of expanded connectivity from not only the SACs, but also mitral cells and centrifugal inputs. In the present study, we have shown that presynaptic connectivity made onto newborn granule cells by SACs is significantly increased in response to sensory stimulation, suggesting they play a pivotal role in the capacity for postnatal-born neurons to integrate within the intact brain. It will be important for future studies to determine, in detail, how the complete repertoire of presynaptic inputs is tuned by sensory experience. Thus, in addition to increasing the number of newly incorporated granule cells [4], [7], [31], [79], sensory experience recruits additional presynaptic elements that contact each granule cell. Such augmented integration may contribute to the high degree of cellular plasticity in this region of the brain and the improved sensory discrimination observed upon odor enrichment [4].

Our slice explant experiments suggest that synaptic transfer of RV is independent of action potentials, VAMP-mediated synaptic release, and fast glutamatergic neurotransmission and thus reflect changes in physical network connectivity. Indeed, odor stimulation increased both spine density and inhibitory synapses on new granule cells, consistent with an expansion of presynaptic input. It remains to be determined if the circuit changes we observed in our experimental paradigm result in long-term structural changes that persist throughout the life of an animal, or represent transient connections that become pruned or lost in the absence of continued odor experience. Our current method of labeling presynaptic inputs is irreversible, and it will be important for future long-term tracing studies to examine the temporal course of granule cell circuit plasticity in response to odor enrichment or deprivation. Such an analysis may require new technologies for repeated genetic labeling of presynaptic partners onto defined cell populations over time.

Our present study has demonstrated the power of new genetic technologies to define neural circuits *in vivo*. The olfactory system is an unusually plastic region of the mammalian brain, capable of continued addition and removal of cell cohorts, with accompanied synapse and circuit remodeling into adulthood. Our results indicate that sensory experience promotes the synaptic integration of new neurons into extensive, spatially organized, cell type-specific olfactory circuits, providing a tractable *in vivo* model to better understand how activity influences complex neural circuit formation. Moreover, adult-born neurons normally destined for the olfactory bulb can reroute to lesion sites in the neocortex and striatum [80], [81], suggesting that the mechanisms of plasticity innate to this renewable cell type may be harnessed for tissue repair. More broadly, monosynaptic tracing *in vivo* provides a powerful approach to map global connectivity of newly integrated neurons during development, plasticity, and regeneration.

## Methods

All experimental procedures and reagents used for this study were approved by the Institutional Animal Care and Use Committees at Baylor College of Medicine and Duke University Medical Center.

### Expression Plasmid Construction

To generate the pCAG-Rabies G-IRES-TVA construct, the cDNA encoding rabies G was excised from pHCMV-RabiesG [82] and cloned into a modified pCIG backbone [83]. For bi-cistronic expression of the TVA receptor, the TVA cDNA from pCMMP-TVA800 [84] was PCR amplified and cloned downstream of an IRES2 element (Clontech, Mountain View, CA) for insertion 3′ to rabies G. For tri-cistronic expression of Cre recombinase, IRES-Cre [85] was inserted downstream of rabies G-IRES-TVA to generate pCAG-rabies G-IRES-TVA-IRES-Cre.

### Generation of *ROSA26-stop^flox^-tdTomato* Mice

A 1.6 kb cDNA fragment encoding the tdTomato protein (provided by Roger Tsien, UCSD) was PCR amplified and cloned upstream of the polyA signal sequence of pCRII. The tdTomato-polyA construct was verified by sequencing. We next constructed a shuttle vector by first cloning the tdTomato-polyA cDNA into the pBigT vector [86] and inserting the CAG promoter element using PacI upstream of the loxP-stop-loxP sequence. We then moved the CAG-loxP-stop-loxP-tdTomato-polyA cassette into the pROSA-acceptor targeting plasmid as previously described [87] to generate the ROSA-CAG-loxP-stop-loxP-tdTomato targeting vector. This targeting construct was linearized and electroporated into E14 ES cells [88]. Following selection, clones were picked and screened for the recombined allele by southern blotting. For southern blots, ES cell genomic DNA was digested with EcoRV, transferred, and hybridized with an external probe to the ROSA26 locus. The wildtype allele gave an 11.5 kb fragment, whereas a 5.7 kb band detected the mutant allele. Using standard procedures [89], positive ES cell clones were used to generate gene-targeted mice. The resulting offspring were genotyped by PCR. To detect both the wildtype and targeted alleles, the following PCR primers were designed for multiplexing: *Rosa/01*, 5′-CACTTGCTCTCCCAAAGTCG -3′; *Rosa/02*, 5′-TAGTCTAACTCGCGACACTG -3′; *CAG/02*, 5′- GTTATGTAACGCGGAACTCC -3′. The wild type allele produced a ∼560 bp fragment with *Rosa/01* and *Rosa/02* primers, whereas the mutant allele was detected by a ∼300 bp fragment with *Rosa/01* and *CAG/02* primers.

### In Vivo Electroporation and RV Labeling of Postnatal-Born Granule Cells

Newborn mice were anesthetized by brief hypothermia. Then, 500 nl of 1 µg/µl endotoxin-free plasmid DNA was injected unilaterally into the right lateral ventricle using a Hamilton syringe with a custom beveled 33-gauge needle (Hamilton Company, Reno, NV). Electroporation was carried out by applying multiple voltage pulses across the width of the newborn heads, just posterior to the eyes, using circular 7 mm tweezertrodes and a BTX ECM 830 square wave electroporator (Harvard Apparatus, Holliston, MA). The electroporation parameters included 5 pulses of 150 V for 50 ms each with 950 ms intervals. Immediately after the procedure, electroporated mice were returned to a heated home cage and monitored until recovery. At 30 d following the electroporation procedure, 250 nl (6×10^3^ particles/µl) of pseudotyped SADΔG-EGFP RV was injected into the granule cell layer of the olfactory bulb using glass injection pipettes and a Nanoject II (Drummund Scientific Company, Broomall, PA). We targeted injection at the midpoint of the olfactory bulb 750 µm below the surface of the brain. This yielded a volume of infection spanning ∼300 µm in diameter±200 µm (see Fig. 1e). At 7 d post-infection, olfactory bulbs were dissected and prepared for image analysis.

### Electroporation and Organotypic Slice Cultures

Organotypic brain slices were prepared and cultured as previously described [52] with minor modifications. For *ex vivo* slices, dorsal telencephalic progenitors were labeled by injecting pCAG-Rabies G-IRES-TVA-IRES-Cre plasmid DNA (0.1 mg/ml) diluted in a 0.1% Fast Green solution into the lateral ventricles of decapitated E14.5 *ROSA26-stop^flox^-tdTomato* mouse heads. The solution was delivered with a small glass capillary pipette attached to a Picospritzer II (General Valve Corp., Fairfield, NJ) using five 15-psi pulses lasting 4 ms each. Electric potentials were generated across intact heads using gold-coated electrodes attached to an ECM 830 electroporator with the following parameters: four 100 ms 45 V pulses separated by 100 ms intervals. Immediately after electroporation, brains were dissected, vibratome sectioned at 250 µm, and maintained as interface organotypic cultures prior to fixation and immunohistochemical labeling. For acute olfactory bulb slices, wildtype newborn mice were electroporated as described above with a DNA plasmid encoding EF1α-tdTomato-P2A-Rabies G-IRES-TVA-WPRE-pA. 10 d later, brains were dissected, vibratome sectioned at 250 µm, infected with virus, and maintained as interface organotypic cultures. The next day pharmacological agents including one or more of TTX (1 µM, Sigma), botulinum toxin A (50 nM, Sigma), tetanus toxin (50 nM, Sigma), APV (50 µM, Tocris), or CNQX (50 µM, Tocris) were added to the culture media and re-administered every 24 h for 5 d. Slices were then fixed, imaged, and counted for fluorescently labeled cells.

### Confocal Imaging and Immunohistochemistry

Experimental mice were sacrificed, perfused with 4% paraformaldehyde in phosphate buffered saline, dissected to remove intact brains, and post-fixed for 1 h at 4°C. Brain tissue was embedded in O.C.T and either sectioned to 12 µm on an upright Leica cryostat, or 50–100 µm slices were cut on a cooled stage microtome. Tissue sections were mounted on slides and imaged using an upright Zeiss 510 scanning confocal microscope (Carl Zeiss Inc.). For immunohistochemistry, sections were incubated with blocking solution (10% normal goat serum, 2% BSA, 0.1% Triton X-100 in PBS pH 7.4) and incubated at 4°C for 2 h. Mouse monoclonal anti-calretinin (1∶1500; Millipore, Temecula, CA), rabbit polyclonal anti-GABA_A_ α1 (1∶1000; Covance), rabbit polyclonal anti-GFAP (1∶1500; Abcam, Cambridge, MA), guinea pig polyclonal anti-parvalbumin (1∶500, Millipore, Temecula, CA), monoclonal anti-gephyrin (1∶2000; Synaptic Systems, Germany), or rabbit polyclonal anti-tyrosine hydroxylase (1∶2000; Novus, Littleton, CO) antibodies were diluted in blocking solution and applied overnight at 4°C. The next day, sections were washed 3×15 min each in PBS with 0.1% Triton X-100, followed by 2×15 min in blocking solution. Secondary Alexa-633 goat anti-rabbit IgG, Alexa-647 donkey anti-mouse, or Alexa-633 goat anti-guinea pig antibodies (Invitrogen, Carlsbad, CA) were then added to a final dilution of 1∶500 and incubated for 4 h at 4°C. Sections were then washed 4×15 min each and mounted with DAPI-containing Vectashield mounting medium (Vector Laboratories, Burlingame, CA). Immunoreacted slides were imaged the same as unprocessed tissues.

#### Double immunofluorescent labeling of BrdU and GFP

Thin sections 14–16 µm were cut on an upright Leica cryostat and collected on Superfrost Plus slides. The slides were air dried for 1 h, rehydrated in PBS, and immersed in blocking solution (described above) for 1 h at room temp. Rabbit anti-GFP antibody (1∶500, Molecular Probes, CA) was then applied to slides in blocking solution overnight at 4°C. The next day, slides were washed 3×5 min each in PBS, followed by application of anti-rabbit Alexa 488 (1∶500, Molecular Probes, CA) for 1 h at room temp. Slides were then washed 3×5 min each, post-fixed in 4% PFA for 15 min, rinsed in PBS 3 times 5 min each, immersed in 0.5N HCl at 55°C for 6 min, post-fixed again in 4% PFA for 10 min, then washed 3×5 min each in PBS. Slides were then digested in a proteinase K solution (0.5 mg/ml) at 37°C for 4 min, post-fixed in 4% PFA for 15 min, then washed 3×5 min each in PBS. After the last wash, slides were immersed in blocking solution for 1 h at room temp, replaced with fresh blocking solution containing mouse anti-BrdU (1∶200, Chemicon) and reacted overnight at 4°C. The next day, the slides were washed 3×5 min each, rinsed with blocking solution, then replaced with blocking solution containing anti-mouse Alexa 594 (1∶500, Molecular Probes, CA) for 1 h at RT. Finally, slides were washed 3×5 min each, coverslipped, and imaged for cell counting.

#### 3D fluorescent image reconstruction

To generate three-dimensional images of RV labeled microcircuits in the olfactory bulb, LSM image files comprising 50–75 z-stack image planes from 150 µm cleared brain slices [61] spaced 1.5 µm apart were processed using Amira segmentation and volume rendering software (Visage Imaging, San Diego, CA). Image planes were captured at 20× full field magnification. Isolated presynaptic networks comprising identifiable single source granule cells and their presynaptic SACs were reconstructed. After tracing all EGFP expressing cell types and performing segmentation, EGFP labeled mitral cells and occasional gial cells were masked from the image reconstruction to offer better image resolution and measurement of SAC networks. Due to the uncertainty of origin for all of the fine neurites extending from the imaging field, measurements of SAC networks was constrained to clearly identifiable dendrites.

#### Analysis of dendritic protrusions

To quantify the number of protrusions we observed on the dendrites of postnatal born granule cells following odor enrichment, we sacrificed mice from odor stimulated and control groups, performed intracardial perfusion, then postfixed for 1 h in 4% paraformaldehyde in PBS. Brain tissue was cryoprotected in 30% sucrose/PBS and frozen in O.C.T. Thin sections (20 µm) were cut on a cryostat and mounted on Superfrost Plus slides. Using an upright Zeiss 510 confocal microscope, doubly labeled (granule source cell) dendrites were first identified extending from RV-infected granule cells under 40× magnification. Imaging and analysis for protrusion counts was performed in randomly sampled doubly labeled dendrite segments 25 µm in length within the internal EPL. Upper and lower boundaries of doubly labeled dendritic segments were identified, and multiple confocal image planes were collected 250 nm apart to generate individual 40-plane, 10 µm thick z-stack image files, so as to span the entire thickness of the dendrite. All counts of dendritic protrusions were performed blind to the experimental manipulation. The morphological criteria established for analysis was that protrusion length (axis perpendicular to dendritic shaft) was ≥ to protrusion width. This was determined in 3 dimensions by manually visualizing each plane of the serial z-stacks for each dendrite segment included in analysis. Data were reported as ± SEM of spine number per 25 µm dendrite on granule cells in mice exposed to cycled odorants (odor) compared to non-odor exposed controls. p<0.001, Student's t-test. For display, representative images of dendritic segments used for protrusion counts were generated as maximal Z-stack projections.

#### Analysis of synaptic puncta

All counts of gephyrin positive puncta were performed blind to the experimental condition. To quantify changes in the number of synaptic puncta following odor stimulation, we prepared virally-labeled olfactory bulb tissue as described above for spine analysis. Briefly, 20 µm sections were cut and mounted on slides. Using an upright Zeiss 510 confocal microscope, doubly labeled dendrites were identified extending from RV-infected granule cells under 40× magnification. Upper and lower boundaries of doubly labeled dendritic segments were identified, and multiple confocal image planes were collected 250 nm apart to generate individual 40-plane, 10 µm thick z-stack image files, so as to span the entire thickness of the dendrite. Gephyrin-positive puncta were identified and included in our analysis only if they were clearly resolved within the doubly-labeled granule source cells. This was determined by manually visualizing each plane of the serial z-stacks for each dendrite segment. Puncta were considered colocalized and counted if gephyrin staining was observed in ≥2 independent image planes through the doubly labeled dendrite. Gephyrin positive scaffolds were excluded from analysis if they were ≥3 µm in any dimension. Data were reported as ± SEM of gephyrin positive puncta per 35 µm dendrite on granule cells in mice exposed to cycled odorants (odor) compared to non-odor exposed controls. p<0.02, Student's t-test. For display, representative images of dendritic segments used for gephyrin counts were generated as maximal Z-stack projections.

### Odor Enrichment

Odor enrichment was carried out using similar parameters as previously described [90]. For controlled odor delivery, a liquid-dispensing robot (model 7200; I & J Fisnar, Fair Lawn, NJ), was programmed to cycle through 42 vials containing different odorant mixtures continuously for 4 weeks following electroporation. Odorants were presented for 5 s each, followed by a 1 min clean air exposure at a flow rate of 0.2 L/min. Odorants were diluted in mineral oil according to their individual vapor pressures to give a nominal headspace concentration of 100 ppm. Further flow diluted the odorants to a nominal final vapor phase concentration of ∼10 ppm. For mixture stimuli, compounds were diluted to the same final delivery concentration.

### Slice Electrophysiology

#### Olfactory bulb slice preparation

Mice were euthanized with pentobarbital sodium (40 mg/kg, i.p.) and decapitated after disappearance of corneal reflexes. Brains were rapidly removed into ice-cold dissection buffer containing (in mM): 87 NaCl, 2.5 KCl, 1.25 NaH_2_PO_4_, 25 NaHCO_3_, 75 sucrose, 10 dextrose, 1.3 ascorbic acid, 7 MgCl_2_, and 0.5 CaCl_2_, bubbled with 95% O_2_ and 5% CO_2_. In the same dissection buffer, the olfactory bulb was isolated and sliced coronally at 250 µM using a vibrating microtome (Leica VT1200S). Slices were allowed to recover for 20 min at 35°C in ACSF containing (in mM): 124 NaCl, 3 KCl, 1.25 Na_2_PO_4_, 26 NaHCO_3_, 1 MgCl_2_, 2 CaCl_2_, and 20 D-glucose saturated with 95% O_2_ and 5% CO_2_, ∼310 mOsm, pH ∼7.25, and kept at room temperature until use. Recordings were made in a submersion chamber at 30–32°C in ACSF.

#### Whole cell recordings

Short axon cells or source newborn granule cells were visually identified with IR-DIC optics and then targeted for recordings through either EGFP, or dual EGFP and tdTomato fluorescence, respectively. Patch pipettes were pulled from thick-walled borosilicate glass with open tip resistances of 2–7 MΩ and were filled with (in mM) 120 K-gluconate, 5 KCl, 2 MgCl_2_, 0.05 EGTA, 10 HEPES, 2 Mg-ATP, 0.4 Mg-GTP, 10 creatinine phosphate, pH 7.3, 280–290 mOsm, internal solution for current clamp experiments to characterize evoked action potential firing in short axon cells. For voltage clamp experiments to record mIPSCs in labeled newborn granule cells, the internal solution was (in mM) 89 CsMeS, 46 CsCl, 1 MgCl_2_, 0.16 CaCl_2_, 0.2 EGTA, 15 HEPES, 4 Na-ATP, 0.4 Na-GTP, 15 TEA-Cl, 14 creatinine phosphate, pH 7.3, 315 mOsm. For mIPSC recordings, cells were clamped at −80 mV and bath perfused with 50 µM APV, 50 µM CNQX, and 1 µM TTX. Cells were recorded using a patch clamp amplifier (Multiclamp 700A, Molecular Devices), and data were acquired and analyzed using pCLAMP 10 software (Molecular Devices) and Minianalysis (Synaptosoft).

## Supporting Information

Figure S1 **A Conditional Reporter Allele Combined with Monosynaptic Circuit Tracing.** (a) Diagram of the *ROSA26-stop^flox^-tdTomato* targeting vector. EcoRV digestion was used to identify positive clones by southern blot analysis using a radiolabeled probe to the indicated region. (b) Left, southern blot analysis showing a positively targeted clone indicated by the additional 5.7 kb band produced by introduction of an additional EcoRV site into the targeting vector; right, PCR genotyping data using forward (For) and reverse (Rev) primers as indicated in (a) revealing the presence of the targeted knock-in allele in heterozygous and homozygous *ROSA26-stop^flox^-tdTomato* mice. +, wildtype; tgt, knock-in allele. (c) A cortical slice explant from a *ROSA26-stop^flox^-tdTomato* mouse following electroporation of the rabies-G-IRES-TVA-IRES-Cre construct into the lateral ventricle. Note the high levels of uniform tdTomato expression following Cre introduction. (d) SADΔG-EGFP expression in the same slice shown in (c) three days after RV application. (e) A merged fluorescent image of the conditional tdTomato and SADΔG-EGFP expression shown in (c) and (d). Scale bar, 1 mm. (f–h) A higher magnification view of a trans-synaptically labeled cortical microcircuit. Scale bar, 10 µm. (f) Conditional tdTomato expression in cortical neurons that received the G-IRES-TVA-IRES-Cre expression construct. (g) SADΔG-EGFP expression in a local network of cortical cells trans-synaptically labeled by RV. (h) A merged image showing the originally infected source cell (yellow) and local presynaptic partners (green). (i) A merged image of widespread reporter expression throughout the cortical layers of a recombined and infected slice explant. Note the extensive presynaptic labeling (green) from a limited number source cells (yellow). Arrows identify labeled source cells; L5, layer 5. Scale bar, 10 µm. Analysis of labeled cells was performed in n = 24 slices from 12 embryos.
(TIF)

Figure S2 **Electroporation Targets Postnatal Born Neurons for Stable Plasmid Integration, but not Their Stem Cell Progenitors.** (a) Labeling strategy to determine if granule cells born after electroporation (EP) harbor a stably integrated expression construct. Newborn mice were electroporated with the G-IRES-TVA-IRES-Cre construct and 14 d later treated with BrdU in the cage water for an additional 14 d to label all neurons born thereafter. 28 d after electroporation, mice were injected with SAD**Δ**G EGFP RV in the olfactory bulb, and subsequently processed for dual BrdU and EGFP imaging 1 week later. (b) Coronal slice through the olfactory bulb showing BrdU labeling. (c) SAD**Δ**G EGFP expression in the slice shown in (a). (d) Merged image of (a) and (b). GL, glomerular layer; EPL, external plexiform layer; MCL, mitral cell layer; GCL, granule cell layer. Scale bar, 50 µm. (e) Graph showing the lack of BrdU labeled neurons expressing SAD**Δ**G EGFP, indicating that stable expression of G-IRES-TVA-IRES-Cre does not propagate in stem cell progenitors. ^*^p<0.01, n = 3 bulbs.
(TIF)

Figure S3 **SADΔG-EGFP Expression in Presynaptic Inputs to Granule Cells.** (a)–(e) Brain sections from mice following intraventricular injection and electroporation with rabies-G-IRES-TVA and subsequent infection in the granule cell layer of the olfactory bulb 30 d later with SADΔG-EGFP RV. This approach ensures selective SADΔG-EGFP RV infection of newborn granule cells. (a) SADΔG-EGFP viral vector expression in anterior olfactory nucleus (AON) neurons indicating monosynaptic transfer from infected OB granule cells. tdTomato expression can be observed in the olfactory bulb (OB). Boxed inset corresponds to higher magnification view shown in (c). Scale bar, 300 µm. (b) SADΔG-EGFP viral vector expression in the nucleus of the horizontal limb of the diagonal band nucleus (HDB) and piriform cortex (PCTX) neurons. Left inset corresponds to higher magnification view shown in (d), whereas right inset corresponds to (e). Scale bar, 350 µm. (c) SADΔG-EGFP expression in AON neurons. (d) SADΔG-EGFP expression in neurons of the HDB. (e) SADΔG-EGFP expression in piriform cortex neurons. Scale bars (c)–(e), 50 µm.
(TIF)

Figure S4 **System for Robotic Odor Delivery.** (a) An image of the robotic system designed for cycled forced air odorant delivery. The robot was programmed to continually cycle through multiple vials containing volatile odor compounds for 30 d following electroporation. (b) The list of volatile odor compounds that were repeatedly delivered to mice targeted for monosynaptic tracing of olfactory bulb microcircuits.
(TIF)

Figure S5 **Blockade of Synaptic Activity Does Not Affect Rabies Virus Transfer.** (a)–(e) Monosynaptic labeling in cultured olfactory bulb slices made from mice electroporated *in vivo* with a plasmid encoding tdTomato, Rabies G, and TVA, followed by *in vitro* infection with SAD**Δ**G-EGFP and treatment with pharmacological blockers of synaptic activity. In all slice conditions, granule cells susceptible to RV infection are labeled red, source granule cells are labeled red and green (yellow, arrows), and presynaptic input cells are labeled green. (a) Control slice without pharmacological treatment. Slices treated with botulinum toxin (BoTX, 50 nM) (b), tetanus toxin (TeTN, 50 nM) (c), tetrodotoxin (TTX, 1 µM) (d), or 50 µM CNQX plus 50 µM APV (e). Scale bar, 150 µm. (f) Graph summarizing the average number of presynaptic input cells observed per labeled granule source cell. Data represent means ± SEM of all labeled presynaptic input cells counted in 6 slices for each condition. No significant differences were observed compared to control.
(TIF)
