# Supplementary figures and images for: Activity-Induced Remodeling of Olfactory Bulb Microcircuits Revealed by Monosynaptic Tracing
Source: PLoS One. 2011 Dec 28;6(12):e29423. doi: 10.1371/journal.pone.0029423 (PMC3247270; doi:10.1371/journal.pone.0029423)

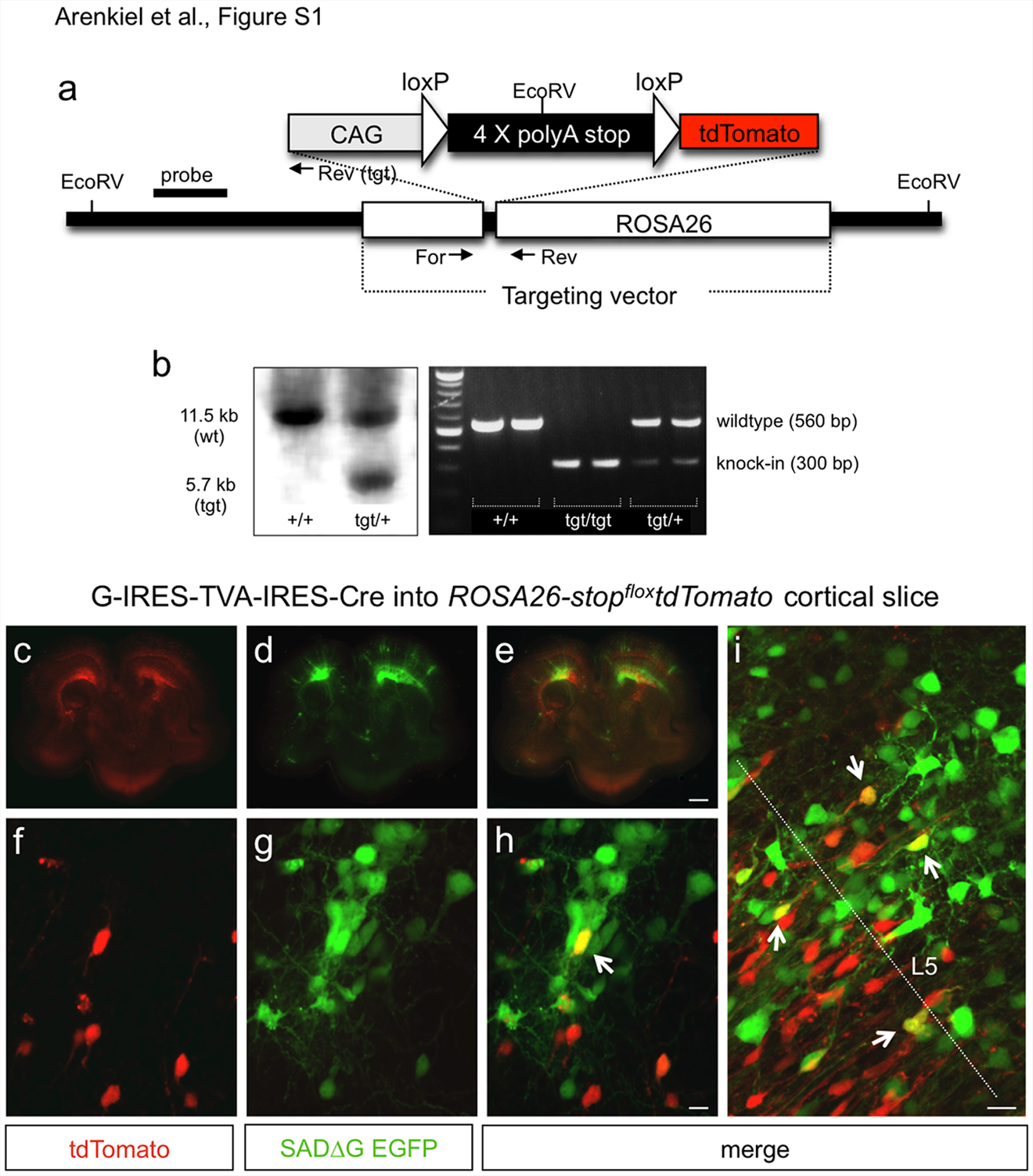

Supplement: Figure S1 — A Conditional Reporter Allele Combined with Monosynaptic Circuit Tracing. (a) Diagram of the ROSA26-stopflox-tdTomato targeting vector. EcoRV digestion was used to identify positive clones by southern blot analysis using a radiolabeled probe to the indicated region. (b) Left, southern blot analysis showing a positively targeted clone indicated by the additional 5.7 kb band produced by introduction of an additional EcoRV site into the targeting vector; right, PCR genotyping data using forward (For) and reverse (Rev) primers as indicated in (a) revealing the presence of the targeted knock-in allele in heterozygous and homozygous ROSA26-stopflox-tdTomato mice. +, wildtype; tgt, knock-in allele. (c) A cortical slice explant from a ROSA26-stopflox-tdTomato mouse following electroporation of the rabies-G-IRES-TVA-IRES-Cre construct into the lateral ventricle. Note the high levels of uniform tdTomato expression following Cre introduction. (d) SADΔG-EGFP expression in the same slice shown in (c) three days after RV application. (e) A merged fluorescent image of the conditional tdTomato and SADΔG-EGFP expression shown in (c) and (d). Scale bar, 1 mm. (f–h) A higher magnification view of a trans-synaptically labeled cortical microcircuit. Scale bar, 10 µm. (f) Conditional tdTomato expression in cortical neurons that received the G-IRES-TVA-IRES-Cre expression construct. (g) SADΔG-EGFP expression in a local network of cortical cells trans-synaptically labeled by RV. (h) A merged image showing the originally infected source cell (yellow) and local presynaptic partners (green). (i) A merged image of widespread reporter expression throughout the cortical layers of a recombined and infected slice explant. Note the extensive presynaptic labeling (green) from a limited number source cells (yellow). Arrows identify labeled source cells; L5, layer 5. Scale bar, 10 µm. Analysis of labeled cells was performed in n = 24 slices from 12 embryos. (TIF) [file pone.0029423.s001.tif]

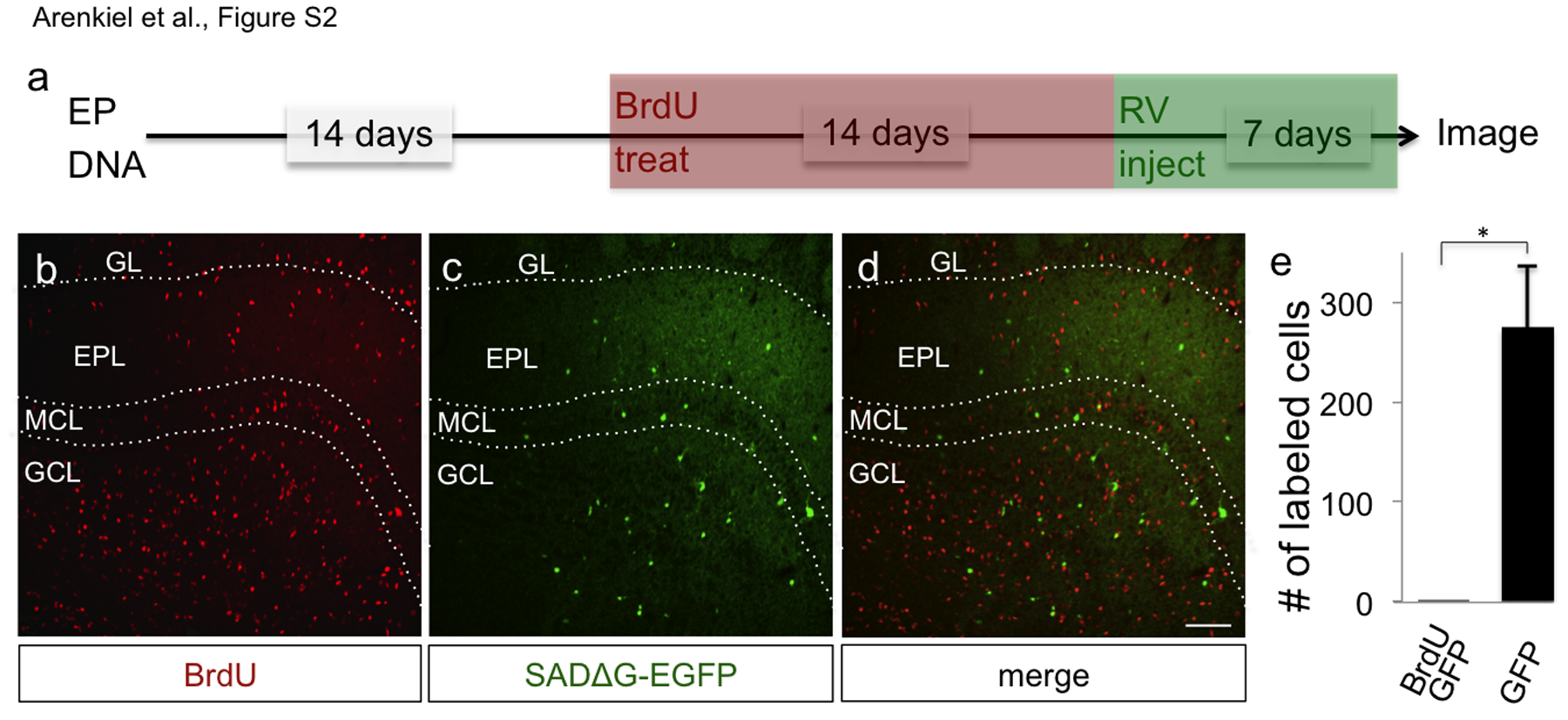

Supplement: Figure S2 — Electroporation Targets Postnatal Born Neurons for Stable Plasmid Integration, but not Their Stem Cell Progenitors. (a) Labeling strategy to determine if granule cells born after electroporation (EP) harbor a stably integrated expression construct. Newborn mice were electroporated with the G-IRES-TVA-IRES-Cre construct and 14 d later treated with BrdU in the cage water for an additional 14 d to label all neurons born thereafter. 28 d after electroporation, mice were injected with SADΔG EGFP RV in the olfactory bulb, and subsequently processed for dual BrdU and EGFP imaging 1 week later. (b) Coronal slice through the olfactory bulb showing BrdU labeling. (c) SADΔG EGFP expression in the slice shown in (a). (d) Merged image of (a) and (b). GL, glomerular layer; EPL, external plexiform layer; MCL, mitral cell layer; GCL, granule cell layer. Scale bar, 50 µm. (e) Graph showing the lack of BrdU labeled neurons expressing SADΔG EGFP, indicating that stable expression of G-IRES-TVA-IRES-Cre does not propagate in stem cell progenitors. *p<0.01, n = 3 bulbs. (TIF) [file pone.0029423.s002.tif]

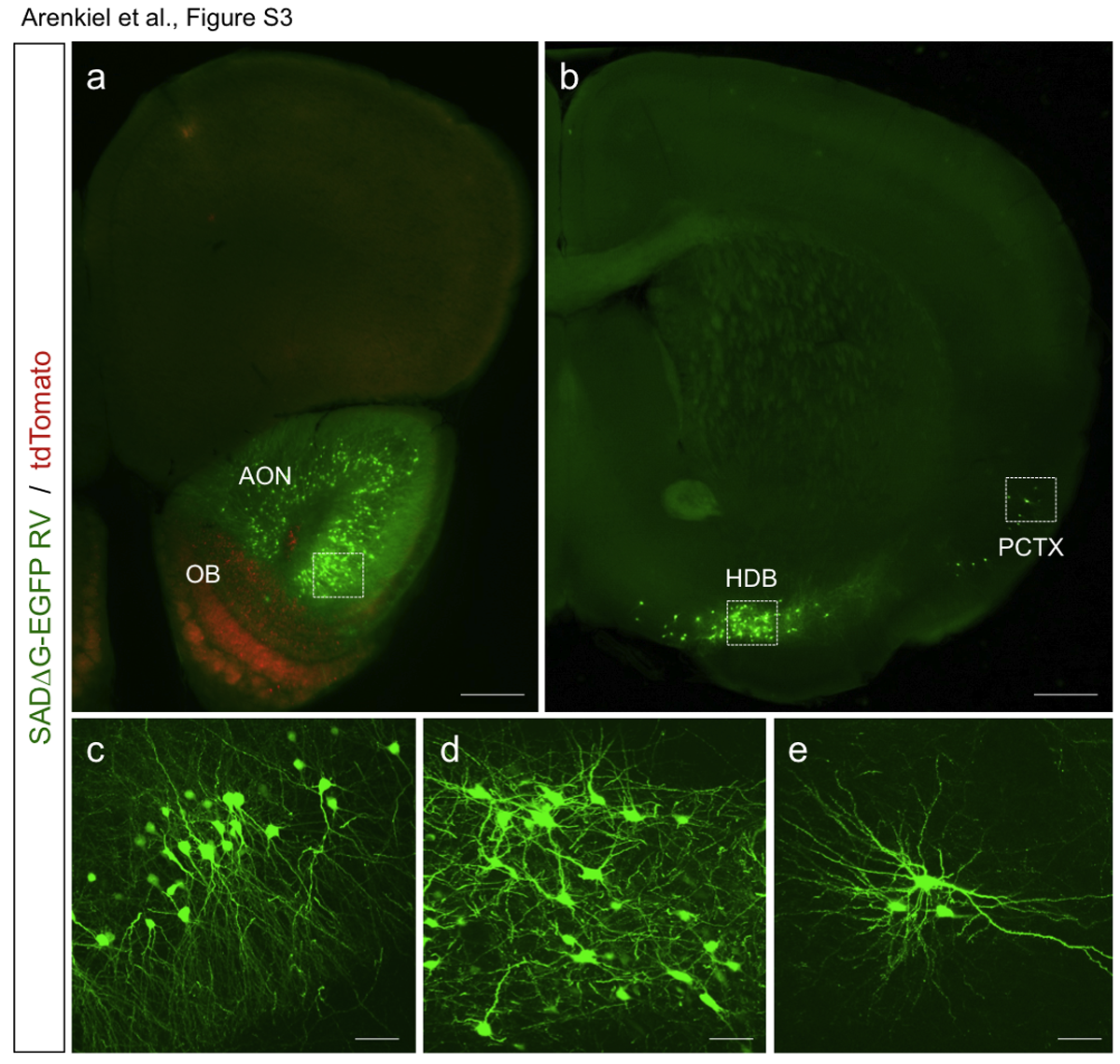

Supplement: Figure S3 — SADΔG-EGFP Expression in Presynaptic Inputs to Granule Cells. (a)–(e) Brain sections from mice following intraventricular injection and electroporation with rabies-G-IRES-TVA and subsequent infection in the granule cell layer of the olfactory bulb 30 d later with SADΔG-EGFP RV. This approach ensures selective SADΔG-EGFP RV infection of newborn granule cells. (a) SADΔG-EGFP viral vector expression in anterior olfactory nucleus (AON) neurons indicating monosynaptic transfer from infected OB granule cells. tdTomato expression can be observed in the olfactory bulb (OB). Boxed inset corresponds to higher magnification view shown in (c). Scale bar, 300 µm. (b) SADΔG-EGFP viral vector expression in the nucleus of the horizontal limb of the diagonal band nucleus (HDB) and piriform cortex (PCTX) neurons. Left inset corresponds to higher magnification view shown in (d), whereas right inset corresponds to (e). Scale bar, 350 µm. (c) SADΔG-EGFP expression in AON neurons. (d) SADΔG-EGFP expression in neurons of the HDB. (e) SADΔG-EGFP expression in piriform cortex neurons. Scale bars (c)–(e), 50 µm. (TIF) [file pone.0029423.s003.tif]

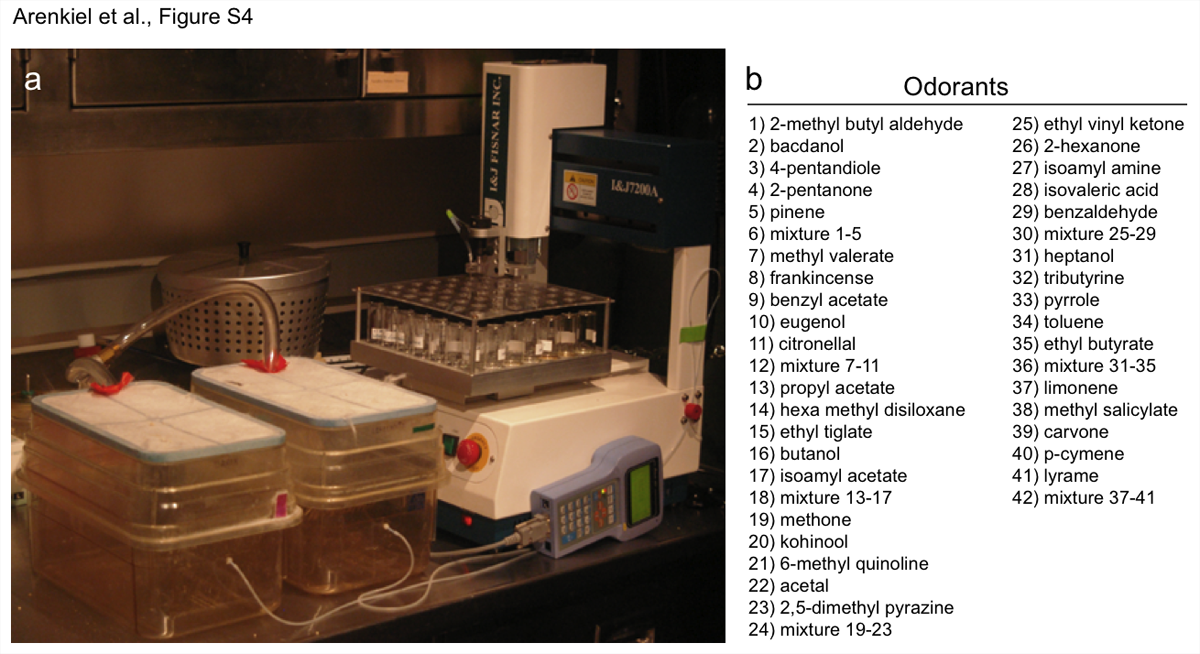

Supplement: Figure S4 — System for Robotic Odor Delivery. (a) An image of the robotic system designed for cycled forced air odorant delivery. The robot was programmed to continually cycle through multiple vials containing volatile odor compounds for 30 d following electroporation. (b) The list of volatile odor compounds that were repeatedly delivered to mice targeted for monosynaptic tracing of olfactory bulb microcircuits. (TIF) [file pone.0029423.s004.tif]

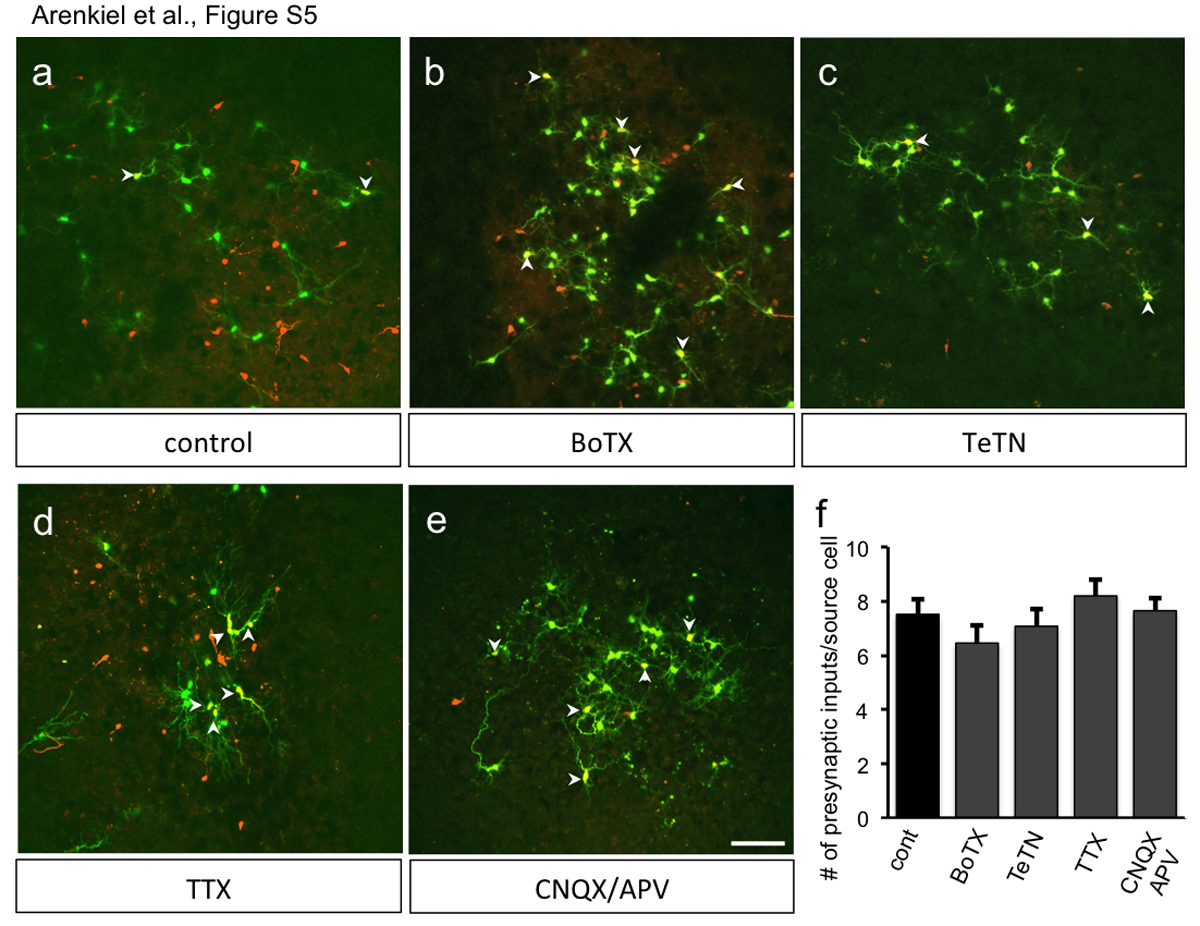

Supplement: Figure S5 — Blockade of Synaptic Activity Does Not Affect Rabies Virus Transfer. (a)–(e) Monosynaptic labeling in cultured olfactory bulb slices made from mice electroporated in vivo with a plasmid encoding tdTomato, Rabies G, and TVA, followed by in vitro infection with SADΔG-EGFP and treatment with pharmacological blockers of synaptic activity. In all slice conditions, granule cells susceptible to RV infection are labeled red, source granule cells are labeled red and green (yellow, arrows), and presynaptic input cells are labeled green. (a) Control slice without pharmacological treatment. Slices treated with botulinum toxin (BoTX, 50 nM) (b), tetanus toxin (TeTN, 50 nM) (c), tetrodotoxin (TTX, 1 µM) (d), or 50 µM CNQX plus 50 µM APV (e). Scale bar, 150 µm. (f) Graph summarizing the average number of presynaptic input cells observed per labeled granule source cell. Data represent means ± SEM of all labeled presynaptic input cells counted in 6 slices for each condition. No significant differences were observed compared to control. (TIF) [file pone.0029423.s005.tif]
